# Perception of Heart Attack Risk Factors and Their Complications Among the Adult Population in the Eastern Region of Saudi Arabia

**DOI:** 10.7759/cureus.49860

**Published:** 2023-12-03

**Authors:** Eman Elsheikh, Abdullah M AlKhudair, Shooq N AlGanem, Hassan R AlDandan, Ali H AlGhareeb, Mohammad A AlSalman, Abdullah A AlKhamis, Hajar M AlHajri, Ghadeer A Alsubaie

**Affiliations:** 1 Department of Cardiology, College of Medicine, Tanta University Hospital, Tanta, EGY; 2 Department of Internal Medicine, College of Medicine, King Faisal University, Al-Ahsa, SAU; 3 College of Medicine, King Faisal University, Al-Ahsa, SAU

**Keywords:** heart attack, risk factors, awareness, myocardial infarction, cardiovascular diseases

## Abstract

Background

Among the young population, many have risk factors that are nonmodifiable, modifiable, or both, increasing their chances of developing cardiovascular diseases (CVDs) and/or experiencing a heart attack later in life. Knowledge of both risk factors has a major role in preventing CVD by encouraging screening and applying the necessary lifestyle modifications. This study aims to assess the knowledge of risk factors and complications associated with heart attacks among individuals in the early adulthood age group.

Methodology

This descriptive cross-sectional study encompassed residents of the Eastern Region who met the specified inclusion criteria. Data were collected and recorded on a structured questionnaire regarding their demographic information, current risk factors, and knowledge of cardiovascular risk factors and complications. The questionnaire was distributed in hard copy among schools, colleges, and primary healthcare centers. The statistical analysis was carried out using IBM SPSS Statistics for Windows, Version 26.0 (IBM Corp., Armonk, NY). The results were presented in tables as percentages and frequencies for all variables, and the scores were presented as mean and standard deviation.

Results

Among the 334 participants, the average overall score was 15.47 out of 28. The participants exhibited varying levels of knowledge across different aspects of the risk factors. The correct response rate ranged from as high as 84.4% (282) to as low as 41.6% (139) in the assessment of risk factors items. The majority of the participants showed a good understanding of the preventive measures, such as exercise and weight reduction. However, 43.4% (145) of participants reported not participating in physical activity and 29.1% (97) had a body mass index of 25 and above.

Conclusions

The outcome of this study suggests that there’s a need for structured educational programs in schools and public health campaigns. The general public must have a reliable source of information rather than the mass media and false information read from social media.

## Introduction

Cardiovascular diseases (CVDs) encompass a range of conditions that primarily impact the heart and blood vessels. These conditions include coronary (ischemic) heart disease (CHD), peripheral arterial diseases, rheumatic heart diseases, congenital heart diseases, cerebrovascular diseases, pulmonary embolism, and deep vein thrombosis [1]. A heart attack, medically known as a myocardial infarction, occurs when a specific area of the heart muscle does not receive an adequate blood supply. This insufficient blood flow is primarily attributed to coronary artery disease (CAD), which is recognized as the main underlying cause. Most heart attacks typically present with discomfort in the center or left side of the chest that persists for more than a few minutes or comes and goes. Additionally, individuals experiencing a heart attack may feel weak or lightheaded, and there may also be the presence of shortness of breath, pain, or discomfort in the jaw, neck, or back, as well as in one or both arms or shoulders. These symptoms collectively serve as warning signs of a possible heart attack [2].

Worldwide, CVD and CHD in particular are major causes of morbidity and mortality. CVDs have emerged as the leading cause of death worldwide. In 2019 alone, an estimated 17.9 million individuals succumbed to CVDs, accounting for a staggering 32% of all global deaths. Among these fatalities, a significant 85% were attributed to heart attacks and strokes. Moreover, in high-income countries, such as Saudi Arabia, ischemic heart disease (IHD) was reported to be the number one cause of death back in 2019. It's estimated that 113.81 deaths per 100,000 people were caused by IHD [3].

With that being said, certain individuals face a higher risk of developing CVD compared to others. Numerous risk factors are associated with CVD, which can be broadly categorized into two groups: modifiable factors and nonmodifiable factors. Modifiable factors include obesity, dyslipidemia, diabetes, hypertension, and smoking, as they can be influenced or managed through lifestyle changes or medical interventions. Nonmodifiable factors, on the other hand, include age, ethnicity, and family history, as they cannot be altered [4,5]. The prevalence of modifiable risk factors is escalating both globally and locally. As an illustration, in Saudi Arabia, it has been reported that 55.5% of the population either have hypertension or falls under the prehypertensive category. Additionally, 20.2% of the population in Saudi Arabia has been diagnosed with diabetes [6,7].

CVD can largely be prevented as the majority of its risk factors, including obesity, smoking, physical inactivity, dyslipidemia, diabetes, and hypertension, are preventable or controllable. Research has provided evidence that the majority of CVDs can be avoided by increasing awareness of primary prevention and secondary prevention measures. This can be achieved through the modification of risk factors such as tobacco use, obesity, physical inactivity, high blood pressure, and unhealthy diet. Additionally, the use of medications like aspirin and blood pressure-lowering agents can also contribute to the prevention of CVDs. By focusing on these preventive measures, individuals can significantly reduce their risk of developing CVDs [8,9]. In addition, adhering to healthy dietary and lifestyle guidelines can significantly contribute to the prevention of CVD [10]. However, although it may seem as simple as this, a crucial part must be taken into consideration. Without perceived knowledge regarding these risk factors and their association with the development of CVDs and CHD in particular, the simple step of modifying these risk factors won’t be taken by the affected individuals. Acquiring knowledge is a crucial step toward adopting a lifestyle promoting cardiovascular health [11].

Research conducted in Riyadh, Saudi Arabia, in 2020, found that 47.1% of the participants had a satisfactory understanding of CVD and its related risk factors. However, there was a lack of awareness regarding the symptoms of stroke and heart attack. Among the respondents, the most commonly recognized symptom of a heart attack was experiencing difficulty in breathing, known by 54.4% of them [12]. In a separate study conducted in Riyadh, Saudi Arabia, approximately half of the participants (46.6%) demonstrated a good understanding of CVD risk factors. However, a significant portion (67.2%) had poor knowledge in this area [13]. Another study regarding the awareness of modifiable acute myocardial infarction risk factors in Jordan in 2015 revealed that even though the patients had an average of two modifiable risk factors for acute myocardial infarction (AMI) and were knowledgeable about them, there was a notable discrepancy between their perceived risks and the actual risks involved [14]. Two more focused studies on heart attack risk factors were done in 1996 and 1978. The first one showed that among 617 adults in Chicago: 28% of the participants identified cigarette smoking as a risk factor for CVD, while 21% recognized high blood pressure and 13% mentioned cholesterol or dietary fat as potential risks. Surprisingly, half of the respondents did not mention these established risk factors, and only 1% correctly identified all three factors [15]. The second study, focusing on heart attack survivors, found that 66% of participants identified stress or worry as the main cause of heart attacks. Surprisingly, only 22% recognized smoking as a contributing factor (28% among smokers). Additionally, there was no significant difference in smoking cessation rates between smokers who identified smoking as a cause and those who did not (78% versus 73%, respectively) [16].

As is seen from previous studies in different countries, the knowledge concerning CVDs and, more specifically, heart attack risk factors is relatively poor. With that, measuring the perception of heart attack risk factors and its complications is of great significance. However, there have been limited studies conducted in this regard in Saudi Arabia, particularly in the eastern region. Therefore, this study is being carried out to assess the knowledge of cardiovascular risk factors and complications in the eastern region of Saudi Arabia.

## Materials and methods

Study sample and design

A descriptive cross-sectional study was carried out in the Eastern Region of Saudi Arabia from January until the end of April 2023. The target group was any Saudi resident in the Eastern Region. Identifying risk factors in the young population allows for early intervention and prevention strategies, and it may be possible to reduce the likelihood of developing heart disease later in life. Therefore, the age group of the studied population was 18 to 45 years. The estimated minimum number of participants required for this study was determined to be 271, with a margin of error set at 5%, a confidence level of 90%, and an anticipated population size of 1,628,280. This was calculated using the Raosoft program (Raosoft Inc., Seattle, WA). The sampling technique used was convenience sampling. A self-administered questionnaire was distributed in hard copy among schools, colleges, and primary healthcare centers. The total number of participants was 345. Certain exclusion criteria were applied at the time of the study to guarantee the accuracy of the results. The exclusion criteria were as follows: All individuals who were non-Saudi, non-residents of the Eastern Province, younger than 18, or older than 45. After applying the inclusion and exclusion criteria, the remaining number of participants was 334.

Study measures and tools

In this study, we used four structured questionnaires to gather comprehensive information and obtain accurate results. The first questionnaire collected demographic data, including age, gender, place of residence, nationality, marital status, occupation, and educational level. The second questionnaire focused on current diseases and possible risk factors, covering lifestyle patterns, physical activity, smoking, and recording height and weight to calculate the body mass index (BMI). It also gathered information on conditions such as hypertension, diabetes, and dyslipidemia, as well as previous diagnoses of heart attack, stroke, transient ischemic attack, or other cardiovascular diseases. The third questionnaire was a modified version of the Heart Disease Fact Questionnaire (HDFQ), assessing knowledge of CHD and its risk factors. It consisted of true or false questions, with 21 statements related to CHD knowledge and risk factors. Scores were calculated based on the number of correct answers, ranging from 0 to 21. Statements with scores below 70% were considered indicative of low knowledge, while scores of 70% or higher indicated adequate knowledge. The modified HDFQ included questions from the original version developed by Wagner, Lacey, Chyun, and Abbott, with additional questions addressing CHD risk factors identified in the National Cholesterol Education Program, Adult Treatment Plan III (NCEP ATP III) [17-20]. The final questionnaire evaluated knowledge of commonly known complications of heart attacks, including heart failure, cardiogenic shock, heart rupture, mitral regurgitation, arrhythmias, pericarditis, and ventricular aneurysm [2,21]. Together, these questionnaires provided comprehensive insights into the participants' demographic profile, current health status, knowledge of CHD, and awareness of heart attack complications.

Statistical analysis

IBM SPSS Statistics for Windows, Version 26.0 (IBM Corp., Armonk, NY) was used to analyze the data. Descriptive statistics are shown as percentages and frequencies for all variables. The overall scores are presented as mean and standard deviation. The correct responses for each statement of the questionnaire are presented in percentages. The data are arranged and presented in different tables to help in understanding the results and determining the level of knowledge and awareness of the studied population. 

## Results

Among the 334 participants, 155 (46.4%) were male and 179 (53.6%) were female. The majority of the participants were aged from 18 to 20 years (197, 59%), resided in a city (296, 88.6%), and were single (257, 82.3%). When analyzing the level of education, it was observed that the majority had excellent educational backgrounds, with nearly half of them having a university degree or were currently studying at a university (152, 45.5%), while more than half (179, 53.6%) had at least a high school degree. The details of the demographic and educational status of the participants are presented in Table 1. 

**Table 1:** Sociodemographic profile (N = 334).

Variables	*n* (%)
Age (years)	
18 to 20	197 (59)
20 to 29	77 (23.1)
30 to 45	60 (18)
Gender	
Male	155 (46.4)
Female	179 (53.6)
Lives in a	
City	296 (88.6)
Village	38 (11.4)
Social status	
Single	275 (82.3)
Married	56 (16.8)
Divorced or widowed	3 (0.9)
Current and highest level of education	
Primary school	1 (0.3)
High school	179 (53.6)
Middle school	2 (0.6)
University	152 (45.5)
Employment status	
Student	270 (80.8)
Employed	256 (76.6)
Unemployed	14 (4.2)

Analyzing the risk factors for CVDs among the participants, such as weight and smoking, revealed that half of the participants had normal BMIs. Nonetheless, 97 (29.1%) participants collectively had a BMI indicative of overweight classification and above. With that being said, only 189 (56.6%) participants were involved in some type of physical activity, while 145 (43.4%) reported not having any physical activity of any sort. However, looking at the smoking status of the sample studied, only 36 (10.8%) were currently smoking and 19 (5.7%) admitted to smoking in the past. Our study also found that 16 (4.5%) individuals reported having CVD, and 5 (31.25%) of them were noncompliant with their medications (Table 2).

**Table 2:** Participants’ characteristics and cardiovascular risk factors (N = 334). ^*^Past smokers are defined as an adult who has smoked at least 100 cigarettes in his or her lifetime but who had quit smoking at the time of the interview, according to the Centers for Disease Control and Prevention (CDC) [22]. BMI, Body mass index

Variables		*n* (%)
BMI		
Less than 18.5 (underweight)		52 (15.6)
18.5 to 24.9 (normal)		167 (50)
25 to 29.9 (overweight)		58 (17.4)
30 to 34.9 (Obese class I)		23 (6.9)
35 to 39.9 (Obese class II)		8 (2.4)
More than 40 (Obese class III)		8 (2.4)
Not recorded		18 (5.4)
Physical activity		
None		145 (43.4)
Aerobics (walking, running, swimming, cycling, and jump robe)		122 (36.5)
Gym		35 (10.5)
Athlete		32 (9.6)
Smoking		
Nonsmokers		279 (83.5)
Ex-smokers*		19 (5.7)
Current smokers		36 (10.8)
Cardiovascular diseases		
No		319 (95.5)
Yes	Hypertension	5 (1.5)
	Diabetes	8 (2.25)
	Dyslipidemia	2 (0.45)
	Arrhythmias	1 (0.3)
Medication compliance		
No		5 (31.25)
Yes		12 (75)

The knowledge assessment of CVDs’ risk factors and complications revealed that the average overall score was 15.47 ± 5.310 out of 28. The detailed score of each section showed an average of 12.79 ± 4.249 out of 21 for the risk factors section and 2.68 ± 1.739 out of 7 for the complications section. Looking at the correct response rate per statement, the majority of participants demonstrated an adequate level of knowledge of risk factors such as age (246, 73.7%), smoking (282, 84.4%), diet (242, 72.5%), and weight (266, 79.6%). On the other hand, fewer participants showed correct knowledge of the other risk factors such as family history (155, 46.4%), hypertension (207, 62%), dyslipidemia (173, 51.8%), and diabetes (139, 41.6%). Furthermore, it was observed that many participants had a satisfactory understanding of preventive measures, including smoking cessation (267, 79.9%), exercise (276, 82.6%), and stress reduction techniques (248, 85%). However, fewer number of participants recognized the importance of controlling blood pressure, glucose, and lipid levels as preventive measures. As for the complications of myocardial infarction, the participants seem to recognize the importance of rapid medical intervention (256, 76.6%). At the same time, their knowledge of other complications ranged from a maximum correct response of (154, 46.1%) to as little as (45, 13.5%). The detailed correct response rates are presented in Table 3.

**Table 3:** Levels of accurate* knowledge of CHD risk factors and complications (N = 334). ^*^Using correct responses only. CHD, coronary heart disease; HDL, high-density lipoprotein; LDL, low-density lipoprotein

Item’s number	Questionnaire item	*n* (%)
1	A person always knows when they have CHD.	100 (29.9)
2	Having a family history of CHD puts you at risk for developing heart disease.	155 (46.4)
3	The older a person is, the greater their risk of having CHD.	246 (73.7)
4	Smoking is a risk factor for CHD.	282 (84.4)
5	A person who stops smoking will lower their risk of developing CHD.	267 (79.9)
6	High blood pressure is a risk factor for developing CHD.	207 (62)
7	Controlling blood pressure reduces a person’s risk for developing heart disease.	233 (69.8)
8	High cholesterol is a risk factor for developing CHD.	173 (51.8)
9	If your *good* cholesterol (HDL) is high, you are at risk for heart disease.	78 (23.4)
10	If your *bad* cholesterol (LDL) is high, you are at risk for heart disease.	188 (56.3)
11	Eating fatty foods does not affect blood cholesterol levels.	242 (72.5)
12	Being overweight increases a person’s risk for CHD.	266 (79.6)
13	Regular physical activity will lower a person’s chance of getting heart disease.	276 (82.6)
14	Only exercising at a gym or in an exercise class will lower a person’s chance of developing heart disease.	137 (41)
15	Walking and gardening are considered exercise that will help lower a person’s chance of developing heart disease.	263 (78.7)
16	Diabetes is a risk factor for developing CHD.	139 (41.6)
17	High blood sugar makes the heart work harder.	194 (58.1)
18	Controlling blood sugar in diabetics reduces their risk of developing CHD.	184 (55.1)
19	Abdominal obesity (fat belly) is a risk factor for developing CHD.	140 (41.9)
20	Stress may cause an increase in blood sugar, blood pressure, and cholesterol levels.	225 (67.4)
21	Slow deep breaths, counting to 10 before speaking, going for a walk are examples of stress stoppers.	284 (85)
22	After having a heart attack, you are vulnerable to have a heart failure.	134 (40.1)
23	*Irregular heartbeats* is not a complications of heart attacks.	119 (35.6)
24	If you have a heart attack, it’s likely to get a cardiogenic shock and die if the adequate treatment isn’t given fast enough.	256 (76.6)
25	Ventricular aneurysm (blood filled bulge on the wall of the heart’s ventricle) could occur weeks or months after having a heart attack.	65 (19.5)
26	It’s not possible for pericarditis (inflammation of the saclike tissue surrounding the heart) to happen as a result of heart attacks.	45 (13.5)
27	Mitral valve insufficiency is one of the complications of having a heart attack.	116 (34.7)
28	Heart’s wall rupture could happen when having a heart attack.	154 (46.1)

## Discussion

In this study, we aimed to measure the awareness and knowledge regarding the risk factors and possible complications of myocardial infarction. The results showed that the mean score of risk factors knowledge was measured to be 12.79 out of 21. This indicates an unsatisfactory level of awareness among the studied participants. Similar findings were found in multiple studies conducted in different countries [20,23,24,25]. When it came to the knowledge of complications, the participants’ mean score was 2.68 out of 7. This is very suggestive of poor knowledge and understanding of the possible outcomes and adverse effects of myocardial infarction. Fear was recognized as an important psychological factor influencing the motivation to seek medical treatment or to delay it. In a study conducted on the impact of fear on healthcare-seeking behavior, it was found that anxiety, panic, and fear of death were motivating patients to seek medical advice sooner rather than delaying it [26]. Therefore, recognizing the aftermath of heart attacks can influence decisions to seek early medical advice and intervention, as well as knowledge of the risk factors involved.

It is known that CAD can be predicted by establishing risk factors in almost 75% of the population [27]. Our studied group showed good knowledge of some of the known risk factors, including age, smoking, weight, and diet. However, they had poor knowledge of other important risk factors such as hypertension, diabetes, and dyslipidemia. This is similar to another study conducted in Kuwait that also demonstrated poor knowledge of these items [25]. This is also reflected in our results when observing the level of medication compliance among the participants who reported having cardiovascular diseases such as hypertension, diabetes, or dyslipidemia. It is observed that 5 (31.25%) of the 16 (4.5%) participants reported noncompliance with their medication. This is to be correlated to them not recognizing hypertension, diabetes, and dyslipidemia as important risk factors to control for the prevention of CAD. The United States Preventive Services Task Force (USPSTF) recommends screening for hypertension to start at 18 and for diabetes at 35 years [28,29]. However, the failure of the population to recognize these two as important contributors to CHD is expected to impact their opinion on the importance of screening. Similarly, our studied population reported high educational backgrounds, with the majority of them having high school or higher education. This should be proportional to the level of knowledge [11]. In contrast, the overall score of knowledge of both risk factors and complications was an average of 15.47 out of 28, with a few participants recognizing some major risk factors. This could be attributed to the mass media's influence on spreading false information and the failure of the public to identify trustworthy sources of information [30]. This yields the crucial role of appropriate health campaigns and implementation of health knowledge into the school curriculums.

Among the 334 participants, 266 (79.6%) identified weight as a risk factor for CAD, but only 140 (41.9%) recognized abdominal obesity as a risk factor. Additionally, 97 (29.1%) participants had a BMI classified as overweight and above. Nonetheless, a good proportion of them accounting for 189 (56.6%) reported being involved in some sort of physical activity ranging from simple aerobic exercises to being athletes and competing in different sports. This is indicative of the level of awareness of the importance of physical activity among the studied population.

Limitations

The cross-sectional study design, coupled with the use of small convenience sampling, hinders the ability to generalize the findings of this study to the larger population. Furthermore, as the majority of participants were aged 18 to 20 years, the use of the convenience sampling method and self-reported data may have introduced bias. As a consequence, the findings might have overestimated or underestimated the level of awareness and knowledge of the studied population, and the results cannot be generalized.

## Conclusions

The assessment of the overall knowledge of myocardial infarction risk factors and complications revealed an inadequate mean score. While many participants had good educational backgrounds and answered some of the risk factor questions correctly, some still failed to identify significant risk factors such as hypertension, diabetes, and dyslipidemia. Furthermore, poor knowledge of the complications associated with heart attacks was observed. These findings strongly suggest the need for the implementation of educational programs within school curriculums and the initiation of a widespread health campaign emphasizing the importance of obtaining information from reliable sources. Furthermore, we suggest that further research should be done to establish the reasons behind the gaps in knowledge.
